# Prevalence, contributing factors, and economic implications of strokes among older adults: a study of North-East India

**DOI:** 10.1038/s41598-023-43977-z

**Published:** 2023-10-06

**Authors:** Jumi Kalita, Mrinmoy Pratim Bharadwaz, Aditi Aditi

**Affiliations:** 1 Lalit Chandra Bharali College, Guwahati, Assam India; 2 Axtria India Pvt. Limited, Pune, India; 3 Department of Survey Research and Data Analytics, International Institute for Population Sciences, Mumbai, 400088 India

**Keywords:** Health care economics, Public health, Epidemiology, Population screening

## Abstract

Stroke is a significant cause of mortality and disability in India, with its economic impact on the rise. This study aims to investigate the prevalence and factors associated with stroke among the elderly population in seven north-eastern states of India and its economic consequences. Data from the initial phase of the Longitudinal Ageing Study in India (2017–2018) were utilized, and bivariate and multivariate analyses were done. Stroke prevalence (1.53%) was notable among both genders, with approximately 1% in females and 2.3% in males. Individuals with low physical activity, higher socio-economic status, and unemployment faced a higher risk of stroke. Females exhibited a 60% lower likelihood [AOR 0.40; (CI 0.250–0.627)] of stroke compared to males and hypertension was a significant risk factor. Stroke patients incur up to INR 50,000 of financial burden, with a considerable proportion facing disability in comprehension and speech. The economic burden of stroke-related hospitalization was significantly high, emphasizing the need for government-funded health insurance to cover stroke-related medications and reducing out-of-pocket expenses for patients seeking treatment in healthcare facilities. The study highlights the urgency for better schemes to address the growing threat of strokes in the north-eastern parts of India for comprehensively tackling this public health challenge.

## Introduction

Stroke is the second principal cause of death and a major cause of disability^1, 2^. Defined as an episode of acute neurological dysfunction presumed to be caused by ischemia or haemorrhage, persisting ≥ 24 h or until death^3^. It is a complicated illness characterized by various symptoms, including sensory, cognitive, perceptual, behavioural, and motor deficits^4^. The prevalence of stroke has increased by 50 percent over the last 17 years, and at present, 1 in 4 people are at risk of getting a stroke in their lifetime^4, 5^. The chance of stroke occurring at age 55 and above is 1 in 5 for women and 1 in 6 for men^6, 7^. The current prevalence rate in India varies from 44.54 to 150/100000^7, 8^. Stroke affects acute neurological dysfunction, such as motor and cognitive damage, and hampers a patient’s day-to-day life by leading to disability^9^. A Global Burden of Disease study in India found that stroke was alone responsible for 9.4 million fatalities and 28.5 million DALYs lost^10^. Despite the availability of numerous medical innovations, interventions and therapeutic approaches, it continues to be one of the leading causes of disability worldwide^11–13^. Developing countries like India with limited resources face a significant challenge from stroke incidence, resulting in partial or severe disability. Generally, there are no proper institutions to care for disabled post-stroke patients, which results in a high rate of stroke morbidity and mortality^14^. It has significant repercussions for the economy due to the loss of human capital as the patient has to depend on others and may no longer remain employed. At the same time, one needs informal care, which also leads to additional economic losses for the patient and the caregivers, eventually this affects the individual and national income^15, 16^. The severity of the patient's condition and the high amount of cost involvement in stroke draw interest in studying the phenomena of stroke, intending to reduce its prevalence and incident rate. A thorough analysis of the economic effect of stroke is essential because it is known that health-care expenditures exacerbate poverty. As per a study using National Sample Survey (NSS) data in India, around 40 million additional people fall into poverty annually due to out of pocket healthcare expenditures^17^. Studies discuss that most stroke care costs revolve around informal care^18^^,^^19^. The incidence and prevalence of stroke in India are quite variable due to differences in demographic, cultural and genetic factors^20, 21^. Literature shows that age is the most critical risk factor of stroke that cannot be improved, as 95% of stroke cases occur at age 45 and above. Out of this figure, two third cases occur at age 65 and above^7^. Thyroid disorder and high blood pressure are other important risk factor for stroke^6, 22^. Besides these, hypertension, diabetes mellitus, high body mass index, and being overweight or obese are known risk factors^23^. A study identifies electrocardiogram (ECG) abnormality, heart disease of any type, diabetes, smoking, and consumption of alcohol as contributing risk factors^24–26^. Multi-morbidity is also common among patients, especially those aged 65 and above who are affected predominantly by multi-morbidities^27^. Less or no physical activity also contributes to the burden directly^9, 28, 29^. Stroke is leading to a significant increase in disease burden nationally, and the north-eastern states of India are no exception^2^.

These states of India have so many hard-to-reach areas, which affects the quality of public health due to accessibility and availability. Given this and the epidemiological shift that the country is undergoing, studying the disease pattern of this section of the population is crucial. A few studies discuss the risk of stroke in older adults with multi-morbidity in depth^14, 30, 31^, however literature on north-eastern states concerning stroke and the economic burden are still dearth. Studying the stroke and its economic burden in the North-eastern states of India is useful for several compelling reasons. The region showcases distinct socio-economic demographic and disease profiles, characterized by a high prevalence of stroke risk factors and limited access to specialized healthcare services. Additionally, the unique cultural and food^22, 32, 33^ practices in these states, very different from the rest of the country, may influence stroke incidence and management. By investigating stroke risk factors and economic burden in this context, we can facilitate the development of targeted interventions and policies to effectively address the burden of stroke, enhance patient outcomes, and alleviate the economic impact on individuals, families, and the healthcare system in the region. Therefore, the study's primary objective is to study the prevalence of stroke in the states of north-eastern India, the disability post-stroke and further identify the economic burden of stroke. The economic analysis will help to understand the economic effect of stroke on the household and consequently on the nation’s GDP. The policymakers can use the outputs of such analysis and identify different cost factors of this particular disease, and accordingly, preventive methods can be adopted. Such thorough estimation of the total cost incurred by a particular disease is also helpful in formulating healthcare policies^34^.

## Methods

### Data

The study uses secondary data from the first wave of the Longitudinal Ageing Study in India (LASI wave-1) conducted from 2017–2018. LASI is a nationally representative survey conducted by the International Institute for Population Sciences, Mumbai, in collaboration with Harvard T. H. Chan School of Public Health (HSPH) and the University of Southern California (USC) under the supervision of the Ministry of Health and Family Welfare (MoHFW), Government of India^35^. The survey included 42,949 households and interviewed 72,250 individuals from the entire nation. The total sample for the current study was 8513, from 5756 households of the north-east part of India. LASI is a Health and Retirement Study based in India. It is India's first survey which includes nearly all aspects of demographic as well as socio-economic and health-related issues (status/ condition) of the individuals aged 45 and above from all corners of the nation. It is scientific research on adult health issues in India, including socio-economic and psychological aspects of older adults. For the collection of data, it used the multistage stratified cluster sampling method. It applies three-stage sampling for the rural areas and four-stage sampling for urban areas to represent the whole of India properly. This practice gives a balanced representation of each part of the nation.

### Variable description

#### Outcome variables

Stroke is a medical emergency when the blood supply to some part of the brain stops for some reason or when a blood vessel in the brain bursts. It leads to damage or death of parts of the brain. The individual may consequently suffer brain damage or death. Stroke symptoms include difficulty or paralysis of limbs and face, speaking and understanding and several other functional limitations. For the current study, the patients self-reported the occurrence of strokes as 'yes' or 'no'.

#### Key explanatory variable

With a sample of individuals aged 45 and above, this study collects data on various co-morbidities and multi-morbidities of the respondents through three different schedules. For the analysis, the following variables were taken as independent variables by trying to examine if there is any effect of these variables on the occurrence of stroke within the sample.

#### Individual factors

Age was categorised as 45–59, 60–75, and 75 + years. Gender was recoded as male and female.

Educational attainment was classified as no education, 1–4 years taken as less than 5 years, 5–9 years, and 10 years or more. Marital status was recoded as currently married, widowed, and others (divorced/separated/deserted/live-in relationship/never married). The Occupation was divided into primary work, unemployed and seasonal workers.

#### Health factors

Health insurance was coded as yes or no. Behavioural health factors such as alcohol and tobacco usage were both coded as yes and no. Sleeping anomalies may cause different diseases in humans. One of the effects of sleeping disorder is increased stress responsivity among individuals, which again increases the risk of stroke; it was recoded as yes or no. An individual's physical activity also plays a vital role in incurring disease. Physical Activity was categorised as every day, 1 to 4 days a week, 1 to 3 times in a month and never. Hypertension/High Blood Pressure, a medical condition that may cause various severe medical conditions of the individual in the long run, which includes increasing the chance of stroke, was recorded as yes and no. Diabetes, a medical condition that may exist due to the imbalance of insulin in the human body, was also categorised as yes and no.

#### Household factors

The monthly per capita expenditure (MPCE) is defined as total monthly household consumption expenditure divided by household size. It is a summary measure of household consumption grouped in 5 quintiles^35^. The MPCE quintile was divided as poorest, poorer, middle, richer and richest. The social hierarchy of people, caste group, was categorised as scheduled caste [SC], scheduled tribe [ST], other backward class [OBC], and general caste. The place of residence was taken as rural and urban.

The calculation of economic cost of stroke required several independent variables that pertain to inpatient and outpatient care of stroke which were directly taken from LASI India report^35^. The variables were consultation costs, amount incurred for blood tests, surgical charges, nursing home charges, transportation costs, and food as well as food and expenses of the accompanying people.

### Statistical approach

The descriptive statistics method was adopted to represent the sample’s different socioeconomic and demographic characteristics, lifestyle factors, and the individuals' multi-morbidity status. Cross tabulations and Chi-Square tests were done to examine the variation and existence of a correlation between stroke prevalence and different socioeconomic and demographic characteristics, lifestyle factors and multi-morbidity conditions. The prevalence of Stroke (Mi) per 1000 older adults was calculated as

$$M_{i} = \frac{A_{i}}{P_{i}}{\star1000}$$

where, Ai = no of older adults that had ever diagnosed with stroke, Pi = Total no of older adults in the sample.

A multiple binary logistic regression was used to examine the behaviours of different characteristics or risk factors of stroke among older adults. The Binary logistic regression model assumes that observations are independent and that no perfect linearity or multi-collinearity exists^36^. Instead, it assumes a linear relationship between the variables and the logit of the result^37^. The model is

$$logit(Y)=ln\left(\frac{p}{1-p}\right)=b_{0}+b_{1}X_{1}+b_{2}X_{2}+.....b_{k}X_{k}$$

where p is the probability of the event, and b0 is the intercept, b_1_, b_2_, …, b_k_ are the regression coefficients, X_i_ s, i = 1, 2, …, k, are the set of predictors.

To understand the cost of inpatient and outpatient care for stroke patients, we calculated the average cost (Mean) and its variability (Standard Deviation) for both types of hospitalization. This provides a brief and informative summary of the cost data, which is valuable for decision-makers and analysts seeking insights into cost structures in healthcare. For analysis of the data, we have used Stata v.15 software^38^.

### Ethics approval and consent to participate

The Central Ethics Committee on Human Research (CECHR) under the Indian Council of Medical Research (ICMR) provided the ethical approval for conducting the LASI survey. Analyses and methods were carried out in accordance with relevant guidelines and regulations. The survey agencies that conducted the field survey for the data collection have collected prior informed consent (signed and oral) for both the inter- views and biomarker tests from the eligible respondents in accordance with Human Subjects Protection.

## Results

### Correlates of stroke prevalence in north-eastern India

**Map 1:**
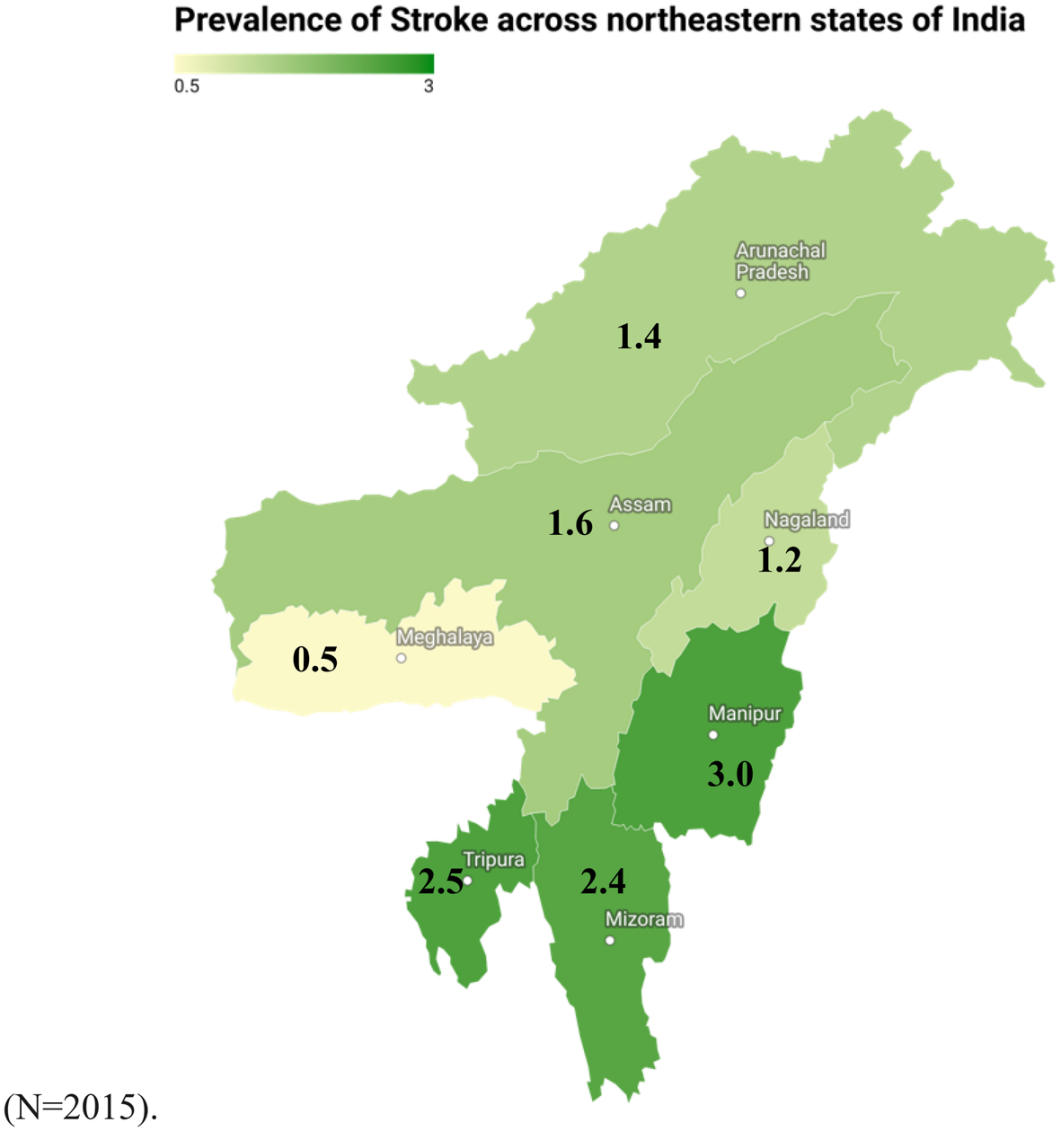
Prevalence of stroke across the seven states of north-eastern India.

The prevalence of stroke across various north-eastern states of India is shown in the Map 1. For the seven states of the country, stroke prevalence is as follows, Arunachal Pradesh 1.4% (N = 982), Nagaland 1.2% (N = 1197), Manipur 3% (N = 1248), Mizoram 2.4% (N = 1128), Tripura 2.5% (N = 1041), Meghalaya 0.5% (N = 885), and Assam 1.6% (N = 2015).

Background characteristics of the eligible respondents are presented in Table 1. The study population included 4070 males and 4426 females in the North-eastern states of India, out of which 130 were diagnosed with stroke. The highest prevalence of stroke (3.69%) was found amongst the age group 75 years and above. While for the age group 45 to 59 years, it was 0.75 percent. Regarding the place of residence, the prevalence of those having a stroke in urban areas (3.26%) was around three times higher than those in rural areas (1.21%). Across castes, general caste people had the highest prevalence of strokes (1.72%) followed by people from the scheduled tribe caste group (1.49%). As per education, the highest prevalence of stroke (2.65%) was found amongst those with the highest education level for 10 or more years. As high as 4 percent of unemployed individuals had a stroke. This was followed by those with seasonal occupation types (0.71%) and those involved in primary work (0.69%). Those from the richest wealth group had the maximum prevalence of having stroke 2.49%. Those who were never involved in any physical activity, the prevalence of stroke was highest amongst them, 2.44%, while those who had some physical activity every day as well as those who had physical activity 1–4 days a week, amongst them, the prevalence of stroke was 0.44% each.

**Table 1:** Demographic variables and its association with stroke patients in North-eastern states of India.

Characteristics	N	Stroke prevalence	Chi-square P-value
Age group			
45–59	4754	0.75	0.000
60–75	2970	2.54	
75 +	772	3.69	
Gender			
Male	4070	2.31	0.000
Female	4426	0.99	
Place of residence			
Rural	6301	1.21	0.000
Urban	2195	3.26	
Caste			
Scheduled caste	684	0.84	0.000
Scheduled tribe	4943	1.49	
OBC	1344	1.46	
General	887	1.72	
Educational status			
No schooling	3493	1.35	0.025
Less than 5 years	1365	1.02	
5–9 years	2168	1.64	
10 or more years	1469	2.65	
Marital status			
Married	6237	1.6	0.555
Widowed	1843	1.81	
Others	416	0.49	
Occupation			
Primary work	1241	0.69	0.000
Unemployed	1792	4.04	
Seasonal workers	3438	0.71	
Wealth quintile			
Poorest	1536	0.42	0.01
Poorer	1638	2.05	
Middle	1672	1.47	
Richer	1746	1.65	
Richest	1904	2.49	
Health insurance			
Yes	2536	1.21	0.840
No	5927	1.91	
Sleep anomaly			
Trouble falling sleep			
Yes	3653	2.26	0.004
No	4838	1.16	
Treatment/medication to sleep			
Yes	127	6.83	0.000
No	8366	1.53	
Alcohol consumption			
Yes	1994	1.63	0.001
No	6389	1.62	
Tobacco consumption			
Yes	4283	1.63	0.047
No	4129	1.60	
Physical activity			
Everyday	2060	0.44	0.000
1 to 4 days a week	1240	0.44	
1 to 3 times in a month	342	2.11	
Never	4827	2.44	
Total	8496	1.53	

### Stroke induced disabilities in north-eastern states of India

Studying the stroke profile of patients from north-eastern states of India, it was found that around 98% of stroke patients were diagnosed by doctors, followed by AYUSH (Ayurveda, Yoga and Naturopathy, Unani, Siddha and Homeopathy) practitioners, 1.6%. More than half of the respondents were on medication for stroke (55.32%), while around 45% still did not take any medicine. Around 26% of the patients undergo any physical or occupational therapy for stroke; however, about two-thirds do not undergo any such therapy. Recurrent occurrence of stroke was seen amongst 24% of the patients. The use of aid or any supportive devices was reported by around 60% (N = 75) of the stroke patients. Amongst those 75 patients, around 34% (n = 29) of the patents were using walking aids/sticks for moving around. Further, the usage of wheelchairs and walking sticks was reported by 9% (n = 7) of the patients among stroke patients who used supportive devices. Around 62% of stroke patients consulted doctors in the last 2 years, and more than half of the stroke patients faced difficulty in speaking or moving due to stroke and suffered from physical disabilities due to stroke. Speech difficulties and inability to think or say the right words were seen among 57% of the patients. Around 19% of the patients suffered from diabetes, as high as 80% from hypertension, and 5% had higher cholesterol (Table 2).

**Table 2:** Stroke profile of the patients from North-eastern States of India, LASI (2017-18).

Determinant (N = 130)	N	Distribution (%)
Who first diagnosed with stroke		
Doctor	127	98.13
AYUSH*	1	1.65
Others	2	0.22
Stroke medication		
Yes	67	55.32
No	63	44.68
Physical/occupation therapy		
Yes	33	26.00
No	96	73.00
Recurrent occurrence of stroke		
Yes	39	24.17
No	91	75.83
Consulted doctor in last 2 years(N = 39)		
Yes	30	62.17
No	9	37.83
Difficulty in moving/speaking with due stroke?		
Yes	66	54.16
No	64	45.84
Use of supportive device		
Yes	75	59.44
No	55	40.56
Walker (N = 75)		
Yes	50	66.00
No	25	34.00
Wheelchairs(N = 75)		
Yes	68	91.00
No	7	9.00
Adjustable shower stools/commodes (N = 75)		
Yes	68	91.00
No	7	9.00
Back/neck collar (N = 75)		
Yes	75	100.00
No	0	0
Orthosis/Prosthesis (N = 75)		
Yes	75	100.00
No	0	0
Physical disabilities		
Yes	66	54.16
No	64	45.84
Speaking/Swallowing problems (N = 66)		
Yes	30	50.05
No	36	49.95
Difficulty in thinking or finding the right words to say		
Yes	31	57.11
No	35	42.89
Difficulty with vision		
Yes	31	37.08
No	35	62.92
Diabetes		
Yes	31	18.91
No	99	81.09
Hypertension		
Yes	101	80.76
No	29	19.24
Higher cholesterol		
Yes	9	4.87
No	121	95.13

*Ayurveda, Yoga and Naturopathy, Unani, Siddha and Homeopathy.

The results of the multiple logistic regression models describing various cofactors for stroke in north-eastern states of India reveal that the odds of 60–75-year-old people suffering from a stroke was 3.2 [AOR: 3.18, 95% CI 2.070–4.888] times more than those in the age group of 45–59 years. The odds of females suffering from stroke were 60 percent [AOR: 0.40, 95% CI 0.250–0.627] less than males. People from urban areas were 1.8 times [AOR: 1.88, 95% CI 1.280–2.760] more likely to suffer from stroke than those from rural areas. Compared to those who did not get any schooling, those who received schooling for less than 5 years were 17 percent [AOR: 0.83, 95% CI 0.468–1.470] less likely to suffer from stroke. However, for those who received 10 or more years of schooling, they were 31 percent [AOR: 1.31, 95% CI 0.762–2.248] more likely to suffer from stroke. Considering marital status, as compared to married people, widowed were 10 percent [AOR: 1.10, 95% CI 0.685–1.780] more likely to be stroke patients. The odds of richer people suffering from a stroke was 2.04 times [AOR: 2.04, 95% CI 1.002–4.151] more as compared to people from the poorest wealth quintile. Similarly, the odds of suffering from stroke for those from the richest wealth quintile was 2.6 times [AOR: 2.66, 95% CI 1.319–5.347] higher than to the poorest people. Compared to those with trouble falling asleep, those who did not have such a problem were 26 percent [AOR: 0.74, 95% CI 0.513–1.073] less likely to suffer from a stroke. The likelihood of stroke amongst those not undergoing treatment or medication for sleep-related problems was 76 percent [AOR: 0.24, 95% CI 0.117–0.471] less than those undergoing any treatment or medication for sleep problems. The people who had a physical activity every day, compared to those who never had any physical activity, were 4.8 times [AOR: 4.85, 95% CI 2.330–10.088] more likely to have a stroke (Table 3).

**Table 3:** Multivariate logistic regression models describing various cofactors for stroke in North-eastern states of India, LASI (2017-18).

Characteristics	Odds ratio	*p-*value	95% CI
Age group			
45–59®			
60–75	3.18	0.00	(2.070–4.888)
75 +	3.91	0.00	(2.215–6.911)
Gender			
Male^®^			
Female	0.40	0.00	(0.250–0.627)
Place of residence			
Rural^®^			
Urban	1.88	0.001	(1.280–2.760)
Educational status			
No schooling^®^			
Less than 5 years	0.83	0.522	(0.468–1.470)
5–9 Years	0.94	0.822	(0.569–1.564)
10 or more years	1.31	0.329	(0.762–2.248)
Marital status			
Married^®^			
Widowed	1.10	0.685	(0.685–1.780)
Others	0.82	0.697	(0.294–2.269)
Wealth quintile			
Poorest^®^			
Poorer	1.76	0.128	(0.849–3.662)
Middle	1.74	0.135	(0.840–3.620)
Richer	2.04	0.049	(1.002–4.151)
Richest	2.66	0.006	(1.319–5.347)
Health insurance			
Yes^®^			
No	0.83	0.349	(0.552–1.234)
Trouble falling sleep			
Yes^®^			
No	0.74	0.112	(0.513–1.073)
Treatment/medication to sleep			
Yes^®^			
No	0.24	0.00	(0.117–0.471)
Alcohol			
Yes^®^			
No	0.69	0.09	(0.451–1.060)
Tobacco			
Yes^®^			
No	0.91	0.631	(0.613–1.346)
Physical activity			
Everyday^®^			
1 to 4 days a week	1.64	0.316	(0.625–4.283)
1 to 3 times in a month	2.23	0.199	(0.657–7.550)
Never	4.85	0.000	(2.330–10.088)

*CI* confidence interval, ^®^reference category.

*If *p* < 0.05, **if *p* < 0.01, ***if *p* < 0.001.

### Costs incurred by stroke patients

Table 4 represents the costs of inpatient and outpatient care across a variety of categories. In terms of health care provider’s fees, outpatient consultations were less expensive than inpatient consultations (INR 893 ± 630 vs. INR 1693 ± 1145). The cost of hospital medications was substantially greater for inpatients (INR 9736 ± 3468) than for outpatients (INR 1710 ± 944). Similarly, outside medication were more expensive for hospitalized patients than for outpatients (INR 7023 ± 1688 vs. INR 4633 ± 1568). Tests and investigations had comparable costs for inpatients and outpatients (INR 7636 ± 2030 and INR 6866 ± 3588, respectively). However, inpatient charges for hospital/nursing home, operating room, blood/oxygen cylinder, transportation, accompanying person expenses, and other expenditures were typically higher than those for outpatients. Overall, inpatient expenditures (INR 41,272 ± 8473) were significantly higher than outpatient expenditures (INR 17,538 ± 6559).

**Table 4:** Cost of Hospitalization and Outpatient Care for Stroke Patients in North-East India.

Characteristics	Inpatient		Outpatient	
	N	Mean ± SD (in INR)	N	Mean ± SD (in INR)
1. Health care provider’s fees (consultation charges)	16	1693 ± 1145	16	893 ± 630
2. Medicines from hospital	15	9736 ± 3468	9	1710 ± 944
3. Medicines from outside	16	7023 ± 1688	16	4633 ± 1568
4. Tests/investigation	18	7636 ± 2030	10	6866 ± 3588
5. Hospital and nursing home charges including bed charges, food	15	8602 ± 3974	5	3628 ± 3854
6. Operation theatre charges, surgery charges and related expenses	9	2861 ± 1823	0	0 ± 0
7. Blood, oxygen cylinder	8	3517 ± 1360	5	7310 ± 3749
8. Transportation	18	4847 ± 1813	17	3417 ± 1429
9. Expenses of the accompanying person(s) (food/accommodation)	14	2711 ± 760	9	1755 ± 775
10. Expenditure not elsewhere reported (others)	15	392 ± 223	12	247 ± 307
Total expenditure	18	41,272 ± 8473	17	17,538 ± 6559

The socio-economic variation of the overall cost incurred by stroke patients in the north-eastern states of India is shown in Table 5. The accounted medical expenditure in any healthcare institution was INR 49,999 ± 41,996 which is higher than average cost of INR 40,360 at national level^17^. The overhead cost appears to increase with increase in wealth of the family. The female elderly respondents have a higher overhead cost than the males and the young elderly age group of 45–59 has the highest average overhead cost than other age- groups. The cost is also higher in urban respondents than rural respondents. It is observed that, people having health insurance have substantially lower medical cost as compared to who did not have any insurance.

**Table 5:** Socio-demographic variations in the economic burden of stroke patients in the north-eastern states of India, LASI 2017-18.

Characteristics	N	Mean ± SD (in INR)
Overall burden	18	49,999 ± 41,996
Age group		
45–59	8	74,603 ± 54,326
60–75	8	34,717 ± 20,521
75 +	2	26,537 ± 13,565
Gender		
Male	7	39,729 ± 18,124
Female	11	55,160 ± 50,096
Place of residence		
Rural	11	48,549 ± 45,235
Urban	7	52,757 ± 38,705
Educational status		
No schooling		
Less than 5 years	4	35,113 ± 25,069
5–9 years	7	81,355 ± 50,140
10 or more years	18	28,683 ± 23,753
Marital status		
Married	12	57,344 ± 39,722
Widowed	5	22,291 ± 16,980
Others	1	157,000
Wealth quintile		
Poorest	0	
Poorer	4	8468 ± 5360
Middle	5	42,560 ± 40,695
Richer	4	45,711 ± 26,700
Richest	5	88,720 ± 49,337
Health insurance		
Yes	4	23,959 ± 10,581
No	14	63,522 ± 45,850
Trouble falling sleep		
Yes	10	60,679 ± 47,808
No	8	30,857 ± 21,200
Treatment/medication to sleep		
Yes	2	105,226 ± 65,653
No	16	45,764 ± 38,315
Alcohol		
Yes	7	34,560 ± 16,602
No	11	59,221 ± 50,212
Tobacco		
Yes	12	33,483 ± 21,824
No	6	90,211 ± 53,407
Physical activity		
Everyday	0	
1 to 4 days a week	1	77,000
1 to 3 times in a month	0	
Never	16	49,875 ± 42,286

## Discussion

This study examined the prevalence of stroke in people aged 45 and above in the North-Eastern region of India, behaviors of different demographic characteristics and the economic costs associated with strokes. There is a paucity of such studies related to the north-east states of India; to the best of our knowledge, this study represents one of the few efforts^2, 17, 22^ aimed at addressing these gaps and elucidating the responses of diverse demographic variables to stroke incidence within this region of the country.

The study revealed a stroke prevalence rate of 1.5 percent in North-Eastern Indian states. This finding is noteworthy, as it closely aligns with the national prevalence of 1.7 percent, as reported in an LASI study^39^ and another study from India using SAGE data showed a national prevalence of 2 percent^40^. Anand et al. (2001) noted a prevalence of 203 per 100,000 population, equating to approximately one million cases in the country^41^. This disparity may, in part, be attributed to the increased susceptibility of South Asians, including Indians, to strokes due to cardio-metabolic risk factors, also discussed by Sarbjeet Khurana et al. (2018) in their research^8^.

Understanding these demographic risk factors of stroke prevalence is essential for targeted interventions and resource allocation in healthcare systems. The findings showed age, sex, place of residence, education, wealth quintile, physical activity and presence of hypertension or diabetes all linked with stroke prevalence. Previous studies also discuss hypertension or diabetes^7, 26^ sex, age, physical activity^29, 42^ modifiable factors like smoking, poor diet, high total cholesterol, exposure to lead, pollution^1^, education and wealth status^43^ are related to the stroke. Older adults over 60 exhibited higher odds of experiencing strokes than their younger counterparts (aged under 60), with males having a higher stroke prevalence than females^44^. These findings corroborate previous studies by Boehme et al.^26^ and others^4, 26, 45^ which have consistently reported a higher prevalence rate of stroke among older adults and males.

Our findings also show that the rate of occurrence of stroke in urban areas is higher than in rural areas. This observation is consistent with other studies^46^ that have suggested dietary preferences, sedentary lifestyles^47, 48^, and a lack of exercise^7, 49^ in urban populations as potential contributing factors. Conversely, lower stroke prevalence in rural areas could be attributed to limited access to health services^25^, balanced dietary habits, and physically demanding occupations. These findings underscore the importance of addressing urban-specific risk factors such as unhealthy diets and sedentary lifestyles through public health interventions and awareness campaigns. Physical activity emerged as a protective factor against stroke in our study. Individuals who engaged in regular physical activity were less likely to experience stroke^50^. This association supports the importance of promoting physical activity as a preventive measure against stroke. Our results align with similar findings from other studies^2, 26, 48^, highlighting the robustness of this relationship across different populations.

Older adults with sleeping problems are more likely to have a stroke in their lifetime, which supports the findings of other studies significantly correlating sleeping problems with stroke^51–54^. The adjusted regression results do not show a significant association of alcohol and tobacco consumption with stroke. This result is not in line with earlier findings such as that of Global Stroke Factsheet, 2022 study^1^. However, another case–control study shows that alcohol intake has no statistically significant association with stroke^24^. Results show hypertension to be one of the significant risk factors for the occurrence of stroke. Several studies found stroke risk factors, of which hypertension was the leading cause^2, 23, 24, 26, 29^. In a study of fifty years of stroke research in India, it was also mentioned that hypertension is one of the major causes of stroke^6^.

Socioeconomic status emerged as a critical determinant of access to healthcare and post-stroke disability. While individuals from higher wealth quintiles have better access to post-stroke rehabilitation and care, they are also more predisposed to stroke due to sedentary lifestyles, stress, diabetes, and obesity. These findings of descriptive analysis align with the literature, emphasizing the relationship between stroke prevalence and economic status^43, 55^. However, they also shed light on the complex interplay between socioeconomic factors and health outcomes. Tailored interventions that consider the socioeconomic context of individuals are essential for achieving equitable stroke prevention and management.

The average economic burden of stroke for inpatient and outpatient care in North-east Indian states were INR 41,272 ± 8473 and INR 17,538 ± 6559, respectively, which includes direct and indirect costs like medications, operation charges, tests, blood, transportation and other costs. Another Indian study by Rajasulochana and Sekhar^17^ also reported higher costs, with a mean of around INR 41,000 per episode of hospitalization. The study's analyis show that a significant part of the economic burden is associated with hospital admissions and length of stay. This finding is consistent with other studies that discuss a substantial direct and indirect cost associated with stroke and duration of stay^17, 56^. There is a substantial gap between the inpatient and outpatient care costs, with inpatient cost being twice the cost of outpatient care. The higher costs again put pressure on the existing economic burden regarding medical treatments, service providers, hospital stays, attending to patients etc. A more extended patient-stay in the hospital results in higher cost^16, 57^. The burden increases substantially among patients with no health insurance, with a significant gap between those insured (INR 23,959 ± 10,581) and those not insured (INR 63,522 ± 45,850). Previous studies have also conferred that only around 10% of the Indian population has health insurance and has a high burden among the uninsured^58^. Their health costs are majorly out-of-pocket expenses. Stroke survivors continue to incur significantly high costs associated with their stroke because of several follow-up and aided medical requirements^56^. The high costs incurred show that the economic burden of stroke-related hospitalization is worrisomely higher in India; to mitigate this, the government-funded health insurance scheme should include coverage for stroke-related aids^57, 58^. It would help reduce the out-of-pocket expenses patients incur by seeking treatment in private healthcare facilities.

As a preventable condition, stroke calls for comprehensive efforts to address its modifiable risk factors entirely or limit their impact through proper guidance and treatment. Public health initiatives aimed at reducing tobacco use, alcohol consumption, promoting healthy diets, and managing conditions like hypertension and diabetes are essential for stroke prevention. Additionally, strategies to increase physical activity levels and raise awareness about the importance of sleep hygiene can reduce stroke incidence. Given India's large and diverse population, addressing stroke's burden requires a multifaceted approach tailored to regional risk factors and healthcare infrastructure variations.

The study's core strength is that it measures and explains the economic burden associated with strokes in the north-eastern states of India based on one of the largest nationally representative ageing surveys. The results call for the need for policymakers in India to increase accessibility and increase the affordability for the socially and economically deprived and vulnerable sections of society. With its lucid analysis and description, the study has meticulously contributed to the current knowledge of the economic burden associated with stroke care in northeast India.

Though the study is a noble attempt to address the gap of knowledge about the prevalence and determinants of stroke in the north-eastern states of India, however, it is not independent of limitations. The data used in the study is cross-sectional. With such data, establishing a robust causal relationship becomes difficult. Hence, future rounds of the LASI data would help to study the causal relation comprehensively. Further, the outcome variables, like the prevalence of stroke and various disabilities following stroke, are self-reported and hence, susceptible to reporting bias. Lastly, other risk factors such as blood pressure levels, obesity and family history of cardiovascular diseases, if available, could further strengthen the results of the current study.

## Conclusion

The prevalence of stroke was found to be around 1.53 percent in the north-eastern states of India. This is a considerable problem among the inhabitants of these states of the country. The study shows that risk factors like age, socio-economic status, level of physical activity and sleep problems are leading causes of stroke in north-eastern states of the country. Sedentary lifestyle habits predispose patients towards several morbidities and subsequent mortality. Stroke is accompanied by various types of disabilities, and mental instability, such as difficulty forming sentences in mind to speak, is the most prevalent. The economic burden of stroke-related hospitalization the states of the north-eastern region is substantial and poses a significant challenge to healthcare systems, necessitating strategies to reduce costs and improve the efficiency of stroke care. The novel findings of this study would help the health sector policymakers in curating policies for the prevention of stroke in patients and their rehabilitation. Measures should be made available to identify the modifiable risk factors of stroke in patients and help them lower the stroke prevalence rate. To meet the needs of stroke survivors, provision of care services either free of cost or at minimal prices should be made available by the government.

## Data Availability

The study uses data from LASI Wave-1 data collected by the nodal agency International Institute for Population Sciences (IIPS), Mumbai, on behalf of the Government of India. Data were de-identified which is publicly available to the researchers and policymakers upon formal request to the nodal agency IIPS. To access the data, a request can be made via this link- https://iipsindia.ac.in/sites/default/files/LASI_DataRequestForm_0.pdf and should be sent to the mail datacenter@iipsindia.ac.in. Further, for information related to the LASI data set Longitudinal Ageing Study in India (LASI), the website of International Institute for Population Sciences (IIPS) (iipsindia.ac.in) can be visited.

## Abbreviations

AOR: Adjusted odds ratio
AYUSH: Ayurveda, yoga and naturopathy, unani, siddha and homeopathy
CI: Confidence interval
DALY: Disability-adjusted life years
ECG: Electrocardiogram
GDP: Gross domestic product
HDL: High-density lipoproteins
LASI: Longitudinal ageing study of India
LDL: Low-density lipoproteins
MoHFW: Ministry of health and family welfare
OBC: Other backward castes
SC: Scheduled castes
ST: Scheduled tribes
